# Size-Dependent Bioactivity of Silver Nanoparticles and Calcium Hydroxide Mixtures Against hDPSCs: An In Vitro Study

**DOI:** 10.3390/ijms262110604

**Published:** 2025-10-31

**Authors:** Ghazal Fakeeha, Lama Al-Zamil, Manikandan Muthurangan, Sayed Auda, Hanan Balto

**Affiliations:** 1 Department of Restorative Dental Sciences, College of Dentistry, King Saud University, Riyadh 11595, Saudi Arabia; 442204701@student.ksu.edu.sa; 2 Department of Clinical Laboratory Sciences, College of Applied Medical Sciences, King Saud University, Riyadh 12372, Saudi Arabia; lalzamil@ksu.edu.sa; 3 Stem Cell Unit, Department of Anatomy, College of Medicine, King Saud University, Riyadh 12372, Saudi Arabia; mrangan@ksu.edu.sa; 4 Department of Pharmaceutics, College of Pharmacy, King Saud University, Riyadh 12372, Saudi Arabia; sauda@ksu.edu.sa

**Keywords:** silver nanoparticles, calcium hydroxide, intracanal medicament, biocompatibility, cell viability

## Abstract

This study aimed to assess the biocompatibility and bioactivity of three different silver nanoparticles (AgNPs) and calcium hydroxide [Ca(OH)_2_] mixtures against human dental pulp stem cells (hDPSCs). hDPSCs were treated with one of the following medicaments: 2 nm mixture, 5 nm mixture, 10 nm mixture, Ca(OH)_2_ alone, and triple antibiotic paste (TAP). Cell viability was evaluated using the Cell Counting Kit-8 and LIVE/DEAD Viability/Cytotoxicity Kit. Reactive oxygen species (ROS) were quantified using the 2′,7′-dichlorofluorescein diacetate redox probe. Transforming growth factor (TGF)-β1, interleukin (IL)-1β, tumor necrosis factor (TNF)-α>, and alkaline phosphatase (ALP) were quantified using enzyme-linked immunosorbent assays. Mineralization was assessed using Alizarin Red S staining. Data were compared across groups using the Kruskal–Wallis test and within groups using the Wilcoxon signed-rank test (*p* < 0.05). Ca(OH)_2_ alone and the 10 nm mixture demonstrated the highest cell viability and lowest ROS release (*p* < 0.05), while the 2 nm and 5 nm mixtures resulted in decreased viability and significant morphological distortion of the cells. Ca(OH)_2_ alone and the 10 nm mixture comparably demonstrated the highest production of anti-inflammatory cytokine TGF-β1 (*p* < 0.05), the lowest production of proinflammatory cytokines IL-1β and TNF-α (*p* < 0.05), and the highest ALP release and mineralization (*p* < 0.05). Within the limitations of this in vitro study, Ca(OH)_2_ alone and the 10 nm mixture improved hDPSCs’ viability, proliferation, differentiation, and mineralization. Both illustrated a significantly higher anti-inflammatory response by the residing stem cell population.

## 1. Introduction

Regenerative endodontic treatment (RET) has emerged as a biologically based treatment strategy to resolve apical periodontitis, resume root maturation, and restore pulpal physiologic conditions [1]. The outcome of this treatment depends on eliminating bacteria and their by-products, which cannot be accomplished only by the chemo-mechanical preparation measures [2]. Therefore, intracanal medicaments are indicated [3]. The American Association of Endodontists (2021) [4] recommends using calcium hydroxide [Ca(OH)_2_] or a lower concentration of triple antibiotic paste (TAP; 1–5 mg/mL) for 1–4 weeks to achieve the intended disinfection. However, the limited antibacterial effectiveness of Ca(OH)_2_ against persistent strains, such as *Enterococcus faecalis* (*E. faecalis*), and TAP’s cytotoxicity highlights the need for alternatives [5].

Incorporating nanoparticles (NPs) into disinfection strategies has gained special attention due to their physiochemical characteristics compared to their bulky counterparts. Silver nanoparticles (AgNPs) were proposed as a promising antibacterial agent in endodontics due to their broad-spectral activity and lower risk of bacterial resistance. Moreover, their application as an intracanal medicament was suggested for the prolonged interaction time required for effective disinfection [6]. Interestingly, combining AgNPs and Ca(OH)_2_ exhibited synergistic activity [7]. This mixture achieved the highest antibacterial efficacy against *E. faecalis* when applied for 7 days [8]. It was also more effective in eradicating *Fusobacterium nucleatum* (*F. nucleatum*) than either medicament alone [9]. Recently, a 10 nm AgNPs + Ca(OH)_2_ mixture demonstrated superior and comparable antibiofilm activity against *Actinomyces naeslundii* (*A. naeslundii*) and *F. nucleatum* to Ca(OH)_2_ and TAP, respectively [10]. Collectively, this suggests the promising application of the mixture in RET.

However, an intracanal medicament should preserve the viability of the residing stem cells [11]. The application of AgNPs in tissue regeneration and repair has been met with reservations due to their cytotoxic/adverse effects on the differentiation potential of different stem cell populations [12]. AgNPs attenuated the adipogenic and osteogenic differentiation of human mesenchymal stem cells (MSCs), even at sub-lethal concentrations [13]. The cytotoxicity of AgNPs is primarily due to the production of reactive oxygen species (ROS) [14]. Interestingly, this cytotoxicity appears size-dependent, where smaller NPs elicit stronger adverse effects [15,16]. Cytotoxicity was greater with 5 nm AgNPs than with 20 and 50 nm AgNPs in four cell lines [15]. ROS production by macrophages was tenfold greater with 15 nm AgNPs than with 30 and 55 nm AgNPs [16]. Moreover, AgNPs significantly upregulate the expression of multiple proinflammatory cytokines in different cell lines [17,18]. Recently, a 15 nm AgNPs gel exhibited comparable cytotoxicity to Ca(OH)_2_ against human dental pulp stem cells (hDPSCs) [19].

The biocompatibility of AgNPs of various sizes and their mixtures with Ca(OH)_2_ towards hDPSCs remains unexplored. The present study tested the hypothesis that smaller AgNPs would exert greater cytotoxic effects on hDPSCs when compared to their larger counterparts. Therefore, this study aimed to explore the size-dependent impact of AgNPs and Ca(OH)_2_ mixtures on hDPSCs’ bioactivity, including cell viability, induced oxidative stress, provoked inflammatory response, odontogenic differentiation, and mineralization.

## 2. Results

### 2.1. hDPSCs Characterization

**Figure 1:**
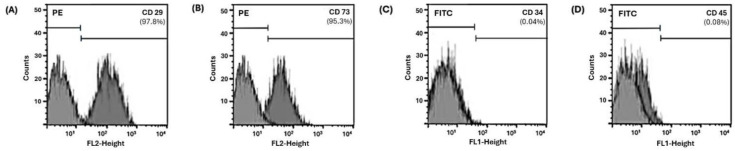
Flow cytometric analysis is presented as histograms that show cell fluorescence intensity on the horizontal axis and cell frequency distribution on the vertical axis. Percentages (%) reveal the expression profile of each surface marker: (**A**) CD29, (**B**) CD73, (**C**) CD34, and (**D**) CD45 against a specific isotype immunoglobulin control (IgG2a-PE and IgG1-FITC).

The examined hDPSCs exhibited relatively low positivity of hematopoietic markers (i.e., CD34 and CD45) and high positivity of mesenchymal markers (i.e., CD29 and CD73; Figure 1).

### 2.2. Cell Viability

**Figure 2:**
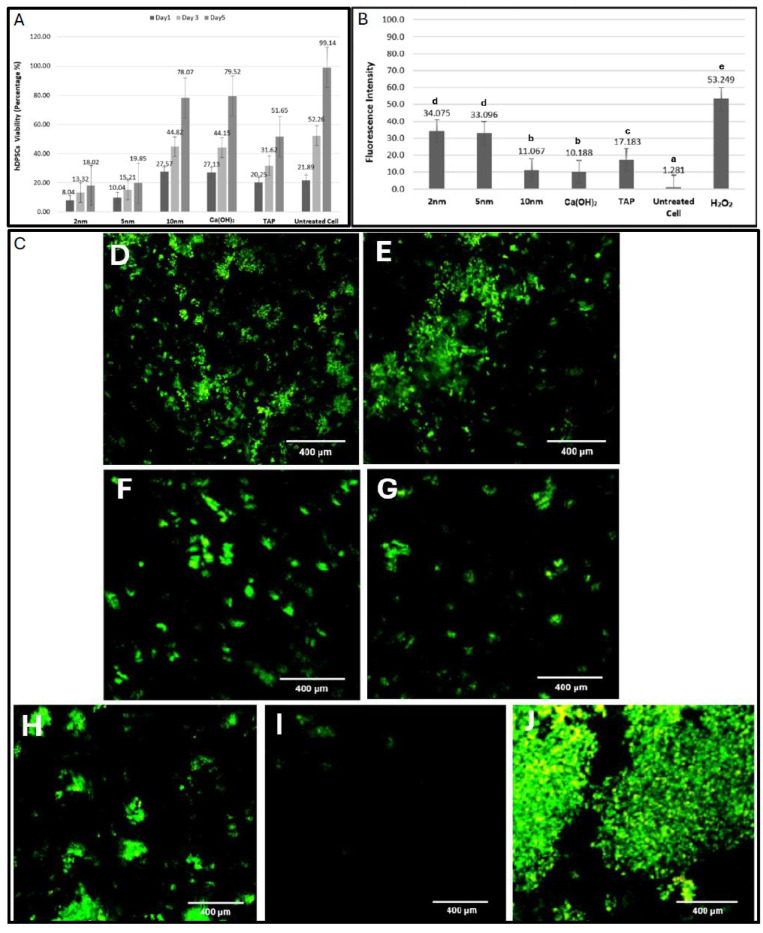
(**A**) Bar graph comparing the % of viable hDPSCs after 1, 3, and 5 days of treatment (data are presented as the mean values of the cellular viability). (**B**) Bar graph comparing the amount of released ROS after 24 h of treatment (different letters indicate a statistically significant difference; *p* < 0.05; data are presented as the mean values of the released ROS). (**C**) A representative inverted fluorescent microscopic image showing intracellular ROS distribution (green) after 24 h of treatment with (**D**) 2 nm mixture, (**E**) 5 nm mixture, (**F**) 10 nm mixture, (**G**) Ca(OH)_2_ alone, (**H**) TAP, (**I**) untreated cells (as a negative control), and (**J**) H_2_O_2_ (as a positive control).

A significant progressive increase in viability was noted among all groups over time (Figure 2A). On day 1, cell viability was greatest with Ca(OH)_2_ alone and the 10 nm mixture (27.13% and 27.57%, respectively; *p* < 0.05) and lowest with 2 and 5 nm mixtures (8.04% and 10.04%, respectively; *p* < 0.05) (Table 1). On day 3, cell viability was greatest with the untreated cells (52.26%), followed by Ca(OH)_2_ alone and the 10 nm mixture (44.15% and 44.82%, respectively). Cell viability was 31.62% with TAP, significantly greater than with 2 and 5 nm mixtures (13.32% and 15.21%, respectively). On day 5, cell viability was still greatest with the untreated cells (99.14%), followed by Ca(OH)_2_ alone and the 10 nm mixture (79.52% and 78.07%, respectively).

**Figure 3:**
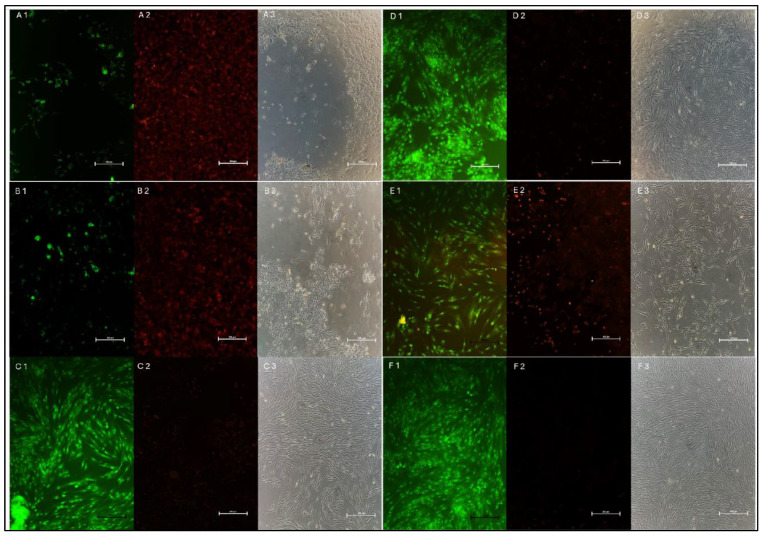
A representative inverted fluorescent microscopic image showing live (green), dead (red), and the general hDPSCs morphological characteristics after 5 days of treatment with (**A1**–**A3**) 2 nm mixture, (**B1**–**B3**) 5 nm mixture, (**C1**–**C3**) 10 nm mixture, (**D1**–**D3**) Ca(OH)_2_ alone, (**E1**–**E3**) TAP, and (**F1**–**F3**) untreated cells (as a control).

The density of live/dead cells was assessed using the green/red fluorescence channels. The findings were consistent with CCK-8 results. The inverted microscopic images of untreated hDPSCs revealed a widely distributed population of typical spindle-shaped cells. Cells treated with Ca(OH)_2_ alone and a 10 nm mixture closely resembled the healthy morphology of the former group. However, TAP exhibited increased spacing between individual cells. 2 nm and 5 nm mixtures exhibited a marked loss of the typical cellular morphology with distinct areas of cellular detachment (Figure 3).

### 2.3. ROS Release

Cells treated with H_2_O_2_ released the highest ROS levels (*p* < 0.05), followed by cells treated with 2 nm and 5 nm mixtures. Ca(OH)_2_ alone and a 10 nm mixture released comparable ROS levels, which were significantly lower than those with TAP. Untreated cells maintained the lowest level among groups (Figure 2B,C).

### 2.4. Proinflammatory/Anti-Inflammatory Cytokine Production

**Figure 4:**
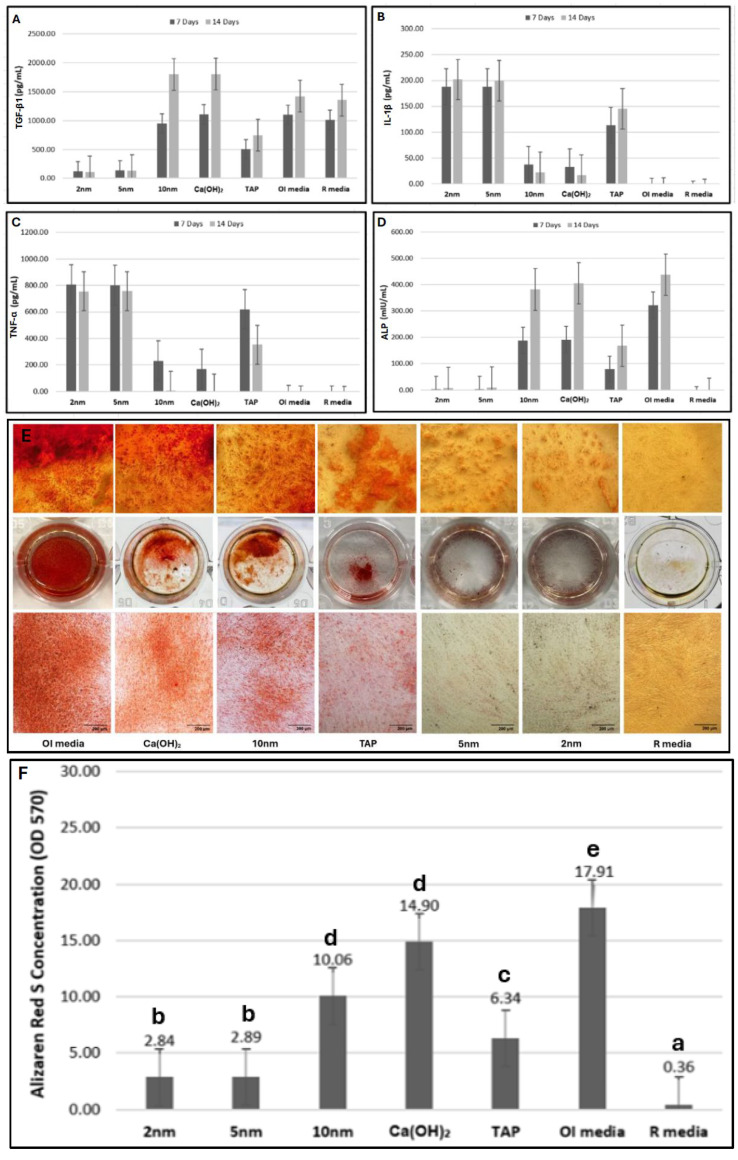
Cytokine levels (pg/mL or mIU/mL) of (**A**) TGF-β1, (**B**) IL-1β, (**C**) TNF-α, and (**D**) ALP after 7 and 14 days of treatment (each column represents the value of the mean). (**E**) a representative inverted light microscopic image of hDPSCs stained with Alizarin Red S after 14 days of treatment, along with a corresponding representative photograph of each culture plate. (**F**) Bar graph representing the absorbance level of the dye retrieved from each plate (different letters indicate a statistically significant difference; *p* < 0.05. Data are presented as the values of the mean).

From day 7 to 14, TGF-β1 production increased significantly with all medicaments except with 2 nm and 5 nm mixtures. On day 14, TGF-β1 production was highest by cells treated with Ca(OH)_2_ alone and a 10 nm mixture (*p* < 0.05). In contrast, TNF-α production decreased significantly over time with all medicaments. At 7 and 14 days, cells maintained in OI and regular medium maintained the lowest cytokine amounts, while 2 nm and 5 nm mixtures produced the highest amounts (*p* < 0.05). Like TNF-α, IL-1β was highest with the former two groups. Ca(OH)_2_ alone and the 10 nm mixture produced similar proinflammatory cytokine amounts of TNF-α and IL-1β (Figure 4A–C; Table 2).

### 2.5. ALP Production

Generally, ALP amounts increased significantly over time and were highest in cells maintained in OI medium, while lowest in cells maintained in regular medium (*p* < 0.05). Ca(OH)_2_ alone and the 10 nm mixture produced comparable ALP amounts, exceeding all other medicaments (Figure 4D; Table 3).

### 2.6. Alizarin Red S Staining

Mineralization was greatest with cells maintained in OI medium (*p* < 0.05), followed by cells treated with Ca(OH)_2_ alone and the 10 nm mixture, then TAP, then the 2 nm and 5 nm mixtures, whereas cells maintained in regular medium induced the lowest mineralization (Figure 4E,F).

## 3. Discussion

RET outcomes depend on the antibiofilm efficacy and biocompatibility of the applied intracanal medicament [11]. In this study, Ca(OH)_2_ and the 10 nm AgNPs mixture showed higher hDPSC viability compared with other medicaments and were associated with better cell proliferation, differentiation, and mineralization potential.

hDPSCs are used as substrates to mimic the clinical situation. hDPSCs are MSCs that can differentiate into osteoblastic, adipogenic, chondrogenic, and odontoblastic-like cells [20]. This population was reported to be positive for mesenchymal markers such as STRO-1, CD29, CD44, CD73, CD90, and CD146, but negative for hematopoietic markers [21]. The flow-cytometric analysis in this study supported this expected profile, though more detailed quantification would strengthen this confirmation. While both hDPSCs and SCAPs are commonly used in RET research, previous work suggests that hDPSCs may provide a more favorable environment for dentin–pulp regeneration due to their stronger expression of odontoblast-related cytokines (Joo et al., 2018), which justifies their selection in the present study [22].

The viability of hDPSCs was assessed after 1, 3, and 5 days of treatment. Previous studies have shown that smaller nanoparticles are taken up by cells more readily than larger ones. The increased cytotoxicity observed with smaller AgNPs is primarily due to the greater release of silver ions, which leads to elevated production of ROS [15,16,23]. Among all tested medicaments, cells treated with 2 nm and 5 nm mixtures demonstrated the lowest viability (18.02% and 19.85%, respectively) and highest ROS production, while those treated with Ca(OH)_2_ alone and the 10 nm mixture demonstrated the greatest viability (79.52% and 78.07%, respectively). After 5 days, 51.65% of hDPSCs treated with TAP remained viable, consistent with multiple studies where Ca(OH)_2_ promoted viability to a greater extent than TAP, especially when the latter was applied at higher concentrations [24,25]. Ruparel et al. reported viabilities of 33–56% for SCAPs treated with 1 mg/mL of TAP, which is comparable to our findings despite the difference in stem cell type [25]. Recently, Saad (2024) reported viabilities of 86.97% and 83.78% for gingival fibroblasts treated with Ca(OH)_2_ alone and an AgNPs + Ca(OH)_2_ mixture, respectively [26]. The higher percentages in his study compared to the current study could be explained by the differences in the applied viability assays and the inherent cell line sensitivity. However, the similar ROS production induced by Ca(OH)_2_ alone and the 10 nm mixture is consistent with his results [26].

It has been established that exposure to AgNPs results in significant production of proinflammatory cytokines and ROS. When oxidative stress is coupled with proinflammatory cytokine release, it produces a positive feedback loop. Therefore, the induced oxidative stress can simultaneously promote the expression of proinflammatory cytokines, which in turn contribute to a stronger oxidative response [17,27]. IL-1β plays a pivotal role in periapical diseases and bone resorption. TNF-α is a potent immunologic mediator of acute/chronic inflammatory responses that can increase the osteoclastic activity [28]. In this study, cells treated with 2 nm and 5 nm mixtures released the highest IL-1β and TNF-α amounts, consistent with other studies, while cells treated with Ca(OH)_2_ alone and 10 nm mixture exhibited the lowest proinflammation [23,29]. Interestingly, Ca(OH)_2_ was found to effectively denature IL-1β and TNF-α, which would explain the lower proinflammatory response reported in the current study [28,30]. In contrast, TAP induced a moderate proinflammation, which was significantly higher than that of Ca(OH)_2_ alone and the 10 nm mixture, consistent with Pereira et al., who found that TAP induced higher IL-1β and TNF-α production than Ca(OH)_2_ in a mouse model [31].

The TGF-β family plays a central role in regulating a broad range of cellular responses, including cell growth and differentiation. TGF-β1 is an anti-inflammatory cytokine that can induce odontoblast differentiation, act as a chemoattractant, and induce matrix synthesis [32]. Depending on the stage of cell maturation, it can either stimulate or inhibit bone formation [33]. Notably, 10 nm AgNPs have been shown to upregulate TGF-β1 signaling in MSCs, enhancing osteogenic differentiation [34]. In our study, Ca(OH)_2_ alone and a 10 nm mixture induced the greatest TGF-β1 production, while TAP maintained lower amounts. This finding is consistent with two studies where greater TGF-β1 amounts were produced by Ca(OH)_2_ than by TAP [32,35]. Ca(OH)_2_ was proven to provide a favorable environment for the anabolic effects of TGF-β1 [36]. The lowest TGF-β1 production by cells treated with 2 nm and 5 nm mixtures could be attributed to the oxidative cellular state, which favors a proinflammatory cellular response rather than repair.

Mineralization potential was assessed via the quantification of ALP production and Alizarin Red S staining. ALP is one of the most frequently used indicators of the osteogenic properties and bone metabolism, and an early-stage odontogenic differentiation marker [37]. After 7 and 14 days of induction in OI medium, Ca(OH)_2_ alone and a 10 nm mixture induced the highest ALP levels and Alizarin Red staining among the tested medicaments. Interestingly, 10 nm AgNPs were found to enhance MSC ALP activity and mineralization after 21 days, with the number of calcium deposits increasing as AgNPs’ concentration was elevated [34]. These findings indicate that the 10 nm mixture can potentially direct hDPSCs toward odontoblastic differentiation and further enhance the mineralization process. The lower mineralization reported in the current study with TAP was consistent with a clinical study that reported higher intracanal calcification with Ca(OH)_2_ (77%) than with TAP (46%) [38]. The high alkaline properties of Ca(OH)_2_ neutralize the acidic environment triggered by inflammatory by-products and therefore enhance mineralization [36]. It is worth noting that the alkaline pH of Ca(OH)_2_ was not significantly altered by the addition of the AgNPs colloidal suspensions while preparing the mixtures, as the pH of the three different mixtures was maintained around 12.5–12.9 [10].

**Figure 5:**
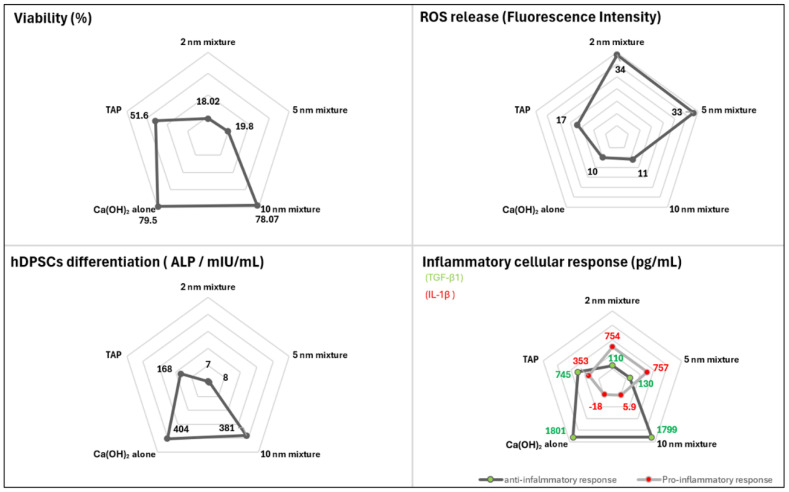
A radar plot summarizing the in vitro performance of the different tested medicaments against hDPSCs: viability after 5 days, ROS release after 24 h, hDPSCs differentiation after 14 days, and the inflammatory cellular response after 14 days of treatment. (ALP: alkaline phosphatase, TGF-β1: transforming growth factor beta 1, IL-1β: interleukin 1 beta).

This study investigated the influence of the incorporated AgNPs’ size on the bioactivity of the residing hDPSCs. Based on its remarkable antibiofilm efficacy [10] and in vitro cell-friendly nature, the data suggest that a 10 nm AgNPs mixture might be a promising alternative to the currently used intracanal medicaments in RET. The clinical relevance of the 10 nm mixture application should be further assessed in an animal model before further clinical exploration. A comparative radar plot illustrating the performance of the different tested medicaments is presented to summarize the findings of the current study (Figure 5).

The commonly recruited stem cells in the RET studies have been from a pulp or an apical origin/location, including hDPSCs from permanent teeth, stem cells of the exfoliated deciduous (SHEDs) teeth, and SCAPs [39]. Nonetheless, hDPSCs may exert a stronger paracrine effect on the dentin–pulp complex regeneration in terms of odontoblast differentiation compared to other available populations [22]. Interestingly, a sub-population of hDPSCs that are CD31− and CD105+ have shown an angiogenic/vasculogenic and neurogenic potential supporting complete pulp regeneration [40]. Several more authors have also successfully implemented this cell population in endodontic regeneration [41,42,43,44,45,46]. Collectively, these reports support the implemented in vitro model in this study to investigate the formerly proposed hypothesis.

Despite the above, numerous phenotypic and MSC-associated marker similarities still exist between DPSCs and SCAPs, making them closely related but histologically distinct [47,48]. Bakopouloua et al. reported that both were able to differentiate into odontoblast-like cells with very active mineralization and migratory potentials in vitro, leading to an organized 3D dentine-like structure. However, SCAPs demonstrated a higher population doubling capacity and proliferation rate, which may be an advantage for dental tissue regeneration applications [48]. Moreover, considering the location of the two former cell lines, Tobias Duarte et al. reported that the apical papilla in immature teeth is more likely to remain viable after inducing endodontic infection, despite the noted pulpal necrosis and the marked apical periodontitis [49,50]. These findings suggest that the apical papilla could provide a relatively stable source and environment for successful RET. Thus, the promising results of the current study could serve as a starting point for future research using SCAPs as an upgraded in vitro model to explore the effect of the 10 nm mixture on the former in order to establish more clinically relevant conclusions. In addition to that, limitations of in vitro models are controversial, and the discrepancies between cell culture biocompatibility findings and clinical data are still documented [51,52,53]. As the in vitro direct physical contact between the applied medicament and the targeted stem cell population does not truly reflect the clinical scenario during RET and therefore could exaggerate the substantial cytotoxicity. Thus, the results of the present study must be interpreted under the highlight of the abovementioned limitations.

## 4. Materials and Methods

This study was approved by the Institutional Review Board of King Saud University (approval number: E-22-7126).

### 4.1. Medicament Preparation

The composition and preparation of each medicament are indicated in Table 4. All medicaments were prepared by a single blinded investigator (G. F) using sterile equipment under a laminar flow cabinet, and the scanning electron microscopy, transmission electron microscopy, and UV spectrophotometry characterization results of each AgNP mixture were previously illustrated [10]. It is worth noting that all medicaments were freshly prepared and used instantly with each carried experiment, while the excess medicaments were discarded and not used in subsequent experiments to ensure the chemical/physical stability of medicaments at the time of application. The data from all carried experiments were collected by a single blinded investigator (M. M) for further analysis and interpretation. A calcium hydroxide viscous vehicle was chosen for the current study to maintain the paste within the well plate for a longer period of time throughout the experiments’ interval and to not be easily washed off by the culture media change [54].

### 4.2. Culturing of hDPSCs

The hDPSCs were generously provided by Dr. Rawan Al-Ateeq, who acquired them from Lonza Bioscience (Catalog #PT-5025; Walkersville, USA). These cells were cultured in α-minimum essential medium (Solarbio, Beijing, China) at a 1:4 split ratio, supplemented with 20% fetal bovine serum (Solarbio) and 1% antibiotic-antimycotic solution (Sigma-Aldrich, Saint Louis, MO, USA)—henceforth referred to as “regular medium”. The cells were maintained at 37 °C with 5% CO_2_ and 95% humidity until 90% confluent. The medium was changed every 3–4 days, and the subsequent experiments utilized cells from passages 4 to 6.

### 4.3. Characterization of hDPSCs

The supplier had characterized the hDPSCs; however, further confirmation was carried out. Single-cell suspensions from passage 4 (1 × 10^6^ cells) were assessed for surface marker expression using flow cytometry with phycoerythrin-conjugated mouse anti-human antibodies against CD29 and CD73 and fluorescein isothiocyanate-conjugated mouse anti-human antibodies against CD34 and CD45 (BD Biosciences, Piscataway, NJ, USA). Experimental cells were incubated with these antibodies for one hour at 4 °C, and control cells were incubated with specific immunoglobulin isotypes per the manufacturer’s instructions. The cell suspensions were analyzed on a FACSCalibur instrument using CellQuest Pro software (version 5.1, BD Biosciences).

### 4.4. Cell Viability

Cell Counting Kit-8 (CCK-8; Abcam, Eugene, OR, USA) was used to quantitatively evaluate hDPSC viability. The cells were incubated overnight in a 96-well plate at a density of 5 × 10^3^ cells/well. After confirming attachment, the cells were treated with 3 µL of the assigned medicament (12 wells/group/observation period). Untreated cells served as the control. The medium was discarded after 1, 3, and 5 days, and cells were rinsed with phosphate-buffered saline. Next, 10 µL of CCK-8 solution and 100 µL of culture medium were added to each well and incubated for 3 h in the dark. The absorbance was measured at 450 nm using a microplate reader (SpectraMax M5 Multi-Mode Microplate Reader; Thermo Fisher Scientific, Waltham, MA, USA). To visualize cell morphology, hDPSCs were stained on day 5 with the LIVE/DEAD Viability/Cytotoxicity Kit for mammalian cells (Invitrogen, Carlsbad, CA, USA) according to the manufacturer’s instructions. Briefly, hDPSCs were incubated with calcein AM (labels live cells green) and ethidium homodimer-1 (labels dead cells red) for 45 min in the dark. Then, images were acquired using excitation/emission wavelengths of 494/517 nm for calcein AM and 517/617 nm for ethidium homodimer-1 with an Eclipse Ti2 inverted fluorescence microscope (Nikon, Shinagawa, Japan).

### 4.5. ROS Release

ROS release was quantified using the 2′,7′-dichlorofluorescein diacetate (DCFH-DA) cell-permeable redox probe (Sigma-Aldrich). Briefly, cells were incubated overnight in a 24-well plate at a density of 3 × 10^5^ cells/well. Next, cells were treated with 10 µL of each medicament (9 wells/group), while untreated cells and cells treated with hydrogen peroxide (H_2_O_2_; 0.2 mol/L) served as negative and positive controls, respectively. After 24 h, cells were washed, and 400 μL of DCFH-DA solution (10 μM) was added to each well and incubated for 45 min in the dark. Then, cells were viewed under an inverted fluorescence microscope using the green fluorescent protein (GFP) channel. To measure the fluorescence intensity, cells were treated with radioimmunoprecipitation assay buffer for 5 min to lyse the cells, and the supernatant was transferred to a black 96-well plate and measured using a microplate reader at an excitation/emission wavelength of 485/530 nm.

### 4.6. Proinflammatory/Anti-Inflammatory Cytokine Production

To assess the inflammatory response, TGF-β1, IL-1β, and TNF-α concentrations were measured using enzyme-linked immunosorbent assays (ELISA; catalog #BMS249-4, BMS224-2, and KHC3011; Invitrogen). Briefly, cells were seeded in a 24-well plate at a density of 3 × 10^4^ cells/well and maintained in an osteoinduction (OI) medium (i.e., regular medium containing 50 µg/mL of ascorbic acid, 10 mmol/L of beta-glycerophosphate, 10 nmol/L of dexamethasone, and 10 nmol/L of vitamin D3) containing 10 µL of the assigned medicament (9 wells/group/observation period). Cells maintained in OI and regular medium served as controls. After 7 and 14 days, supernatants were collected. Dilutions of supernatants and standards for each cytokine were added to the corresponding 96-well plate in triplicate, and the wells were treated per the manufacturer’s instructions. After the ELISA reaction was terminated, the absorbance was measured at 450 nm using a microplate reader, and the standard curve generated for each cytokine was used to determine its concentration in each sample. The results of three independent triplicate experiments were used for analysis.

### 4.7. ALP Production

ALP was measured using ELISA (catalog #EH19RB, Invitrogen) to assess the odontogenic differentiation of hDPSCs. Similar experimental conditions to the former assessment were applied. Cells maintained in OI and regular medium served as positive and negative controls, respectively.

### 4.8. Alizarin Red S Staining

Alizarin Red S staining was used to assess mineralization. After cells were seeded in a 24-well plate at a density of 3 × 10^4^ cells/well, incubation in OI medium + 10 µL of the assigned medicament was implemented (9 wells/group). After 14 days, cells were fixed with 10% formaldehyde and stained with 2% Alizarin Red S (ScienCell Research Laboratories, Carlsbad, CA, USA) for 30 min at room temperature. Then, wells were washed with deionized water and observed using an inverted microscope. The stain was dissolved with 10% (g/v) cetylpyridinium chloride monohydrate (Sigma-Aldrich), and the absorbance was measured at 570 nm using a microplate reader. The results of three independent triplicate experiments were used for analysis.

### 4.9. Statistical Analysis

The data were analyzed using SPSS (version 27; IBM Corp., Chicago, IL, USA). Normality was assessed using the Shapiro–Wilk test, and the data were determined to be non-normally distributed. Accordingly, the data were presented and compared across groups (as the mean ± standard deviation) using the Kruskal–Wallis test, followed by the Mann–Whitney U test with Bonferroni correction, while the Wilcoxon signed-rank test was used across different time points within the same groups (*p* < 0.05).

## 5. Conclusions

The viability and proliferation of hDPSCs in response to the 10 nm AgNPs and calcium hydroxide mixture were comparable to those treated with calcium hydroxide alone. Moreover, the cytotoxic impact of the mixture, as reflected by the level of ROS release, was indistinguishable from that exerted by calcium hydroxide alone. This mixture, along with calcium hydroxide alone, demonstrated a significantly better inflammatory cellular response, differentiation, and mineralization levels than the other tested groups. Within the limitations of this in vitro study, the results suggest that the 10 nm mixture may represent a promising future candidate for clinical use in regenerative endodontics. However, in vivo assessments must be considered first to draw more clinically reliable conclusions.

## Figures and Tables

**Table 1:** hDPSCs viability/proliferation after 1, 3, and 5 days of treatment. Data are presented as the median, mean ± SD of the mean.

Group	Day 1				Day 3				Day 5			
	Median	Mean ± SD (%)	Mean Rank	MCT	Median	Mean ± SD (%)	Mean Rank	MCT	Median	Mean ± SD (%)	Mean Rank	MCT
2 nm	8.89	8.04 ± 4.3	10.96	a °	13.78	13.32 ± 1.8	12.16	a °	18.25	18.02 ± 1	10.46	a °
5 nm	9.98	10.04 ± 3.6	14.04	a °	15.29	15.21 ± 6.4	12.83	a °	19.91	19.85 ± 2.9	14.54	a °
10 nm	27.88	27.57 ± 3.6	64.67	c *	44.76	44.82 ± 1.2	50.17	c ^1^	77.98	78.07 ± 10.5	47.83	c ^1^
Ca(OH)_2_	27.02	27.13 ± 2.2	56.33	c *	44.30	44.15± 4.3	47.33	c ^1^	79.43	79.52 ± 10	49.17	c ^1^
TAP	20.01	20.25 ± 2.5	30.50	b	31.54	31.62 ± 3.8	30.51	b	51.89	51.65 ± 6.3	30.5	b
Untreated Cells	21.77	21.89 ± 0.2	42.50	b	52.07	52.26 ± 5.4	66.01	d *	99.24	99.14 ± 0.5	66.5	d *
*p*-value		0.000				0.000				0.000		

MCT (multiple comparison test). Different letters indicate a statistically significant difference (*p* < 0.05). * (significantly higher viability/proliferation compared to other groups). ° (significantly lower viability/proliferation compared to other groups). ^1^ (significantly lower viability/proliferation than untreated cells but higher than all tested medicaments).

**Table 2:** Pro-/anti-inflammatory cytokine production after 7 and 14 days of treatment. Data are presented as median, mean ± SD of the mean.

Marker	Group	7 Days					14 Days					Wilcoxon Paired Test
		Median	Mean ± SD	Mean Rank	*p* Value	MCT	Median	Mean ± SD	Mean Rank	*p* Value	MCT	
TGF-β1	2 nm	125.32	121.71 ± 20.37	8.50	0.00	a	109.29	110.86 ± 39.05	8.44	0.00	a	0.593
	5 nm	137.41	139.49 ± 37.1	10.50		a	125.49	130.73 ± 35.69	10.56		a	0.859
	10 nm	951.9	953.08 ± 71.25	34.39		c	1811.34	1799.49 ± 92.97	54.22		d	0.008
	Ca(OH)_2_	1118.32	1111.91 ± 49.74	53.72		d	1803.43	1801.97 ± 107.22	54.78		d	0.008
	TAP	502.53	504.98 ± 49.88	23.00		b	741.31	745.24 ± 12.85	23.00		b	0.008
	OI media	1109.01	1101.84 ± 41.82	53.06		d	1430.42	1421.71 ± 53.46	40.00		c	0.008
	R media	1020.21	1013.35 ± 64.81	40.83		c	1355.42	1352.17 ± 51.58	33.00		c	0.008
TNF-α	2 nm	805.54	807.19 ± 4.96	57.00	0.00	d	752.63	754.5 ± 6.73	55.78	0.00	d	0.008
	5 nm	800.43	802.06 ± 6.4	52.00		d	752.21	757.91 ± 27.35	53.22		d	0.021
	10 nm	235.28	231.12 ± 30.36	32.00		b	4.89	5.91 ± 11.06	32.00		b	0.008
	Ca(OH)_2_	171.54	169.31 ± 7.18	23.00		b	−116.87	−18.07 ± 5.06	23.00		b	0.008
	TAP	622.32	619.77 ± 27.89	41.00		c	355.29	353.34 ± 36.84	41.00		c	0.008
	OI media	−101.23	−103.71 ± 1.63	14.00		a	−108.01	−106.32 ± 1.42	13.78		a	0.008
	R media	−105.34	−107.64 ± 0.58	5.00		a	−109.2	−110.06 ± 2.21	5.22		a	0.015
IL-1β	2 nm	190.26	188.27 ± 2.05	56.28	0.00	d	202.11	201.8 ± 4.13	55.44	0.00	d	0.007
	5 nm	189.65	187.95 ± 2.34	52.72		d	120.06	199.59 ± 2.5	53.56		d	0.007
	10 nm	35.01	37.35 ± 1.94	30.00		b	20.87	21.82 ± 1.47	31.56		b	0.007
	Ca(OH)_2_	30.32	32.71 ± 4.23	25.00		b	17.22	16.86 ± 2.23	23.44		b	0.007
	TAP	117.43	113.38 ± 4.54	41.00		c	148.02	145.15 ± 2.43	41.00		c	0.007
	OI media	−24.69	−24.34 ± 0.57	14.00		a	−28.99	−27.86 ± 0.18	12.33		a	0.007
	R media	−25.59	−29.8 ± 2.54	5.00		a	−29.23	−30.24 ± 2.21	6.67		a	0.257

MCT (multiple comparison test). Different letters indicate a statistically significant difference (*p* < 0.05). A red-colored value of the Wilcoxon paired test indicates a statistically significant difference between 7 and 14 days of treatment within the same group.

**Table 3:** ALP levels after 7 and 14 days of treatment. Data are presented as the median, mean ± SD of the mean.

Marker	Group	7 Days					14 Days					Wilcoxon Paired Test
		Median	Mean ± SD	Mean Rank	*p* Value	MCT	Median	Mean ± SD	Mean Rank	*p* Value	MCT	
ALP	2 nm	1.85	1.98 ± 0.78	16.72	0.00	b	7.01	7.06 ± 2.33	16.00	0.00	b	0.008
	5 nm	2.24	2.28 ± 0.26	20.28		b	8.41	8.87 ± 2.95	21.00		b	0.008
	10 nm	188.17	188.19 ± 8.2	44.78		d	382.13	381.25 ± 6.61	43.00		d	0.008
	Ca(OH)_2_	191.88	191.08 ± 6.19	46.22		d	403.98	404.3 ± 3.64	50.00		d	0.008
	TAP	78.88	78.61 ± 16.1	32.00		c	168.62	168.25 ± 17.42	31.00		c	0.008
	OI media	323.22	322.12 ± 13.2	59.00		e	538.21	537.67 ± 13.84	62.00		e	0.008
	R media	−36.78	−36.93 ± 1.19	5.00		a	−32.95	−33.21 ± 1.73	5.00		a	0.011

MCT (multiple comparison test). Different letters indicate a statistically significant difference (*p* < 0.05). A red-colored value of the Wilcoxon paired test indicates a statistically significant difference between 7 and 14 days of treatment within the same group.

**Table 4:** The chemical composition and preparation of each medicament.

Medicament	Chemical Composition	Preparation
2 nm mixture	2 nm AgNPs (0.02%) + 35%Ca(OH)_2_	0.02% of the 2 nm AgNPs colloidal suspension (US Research Nanomaterial, Inc., Houston, TX, USA) was stirred gently and sonicated with 35% Ca(OH)2 paste in a proportion of 1:1, until no lumps observed.
5 nm mixture	5 nm AgNPs (0.02%) + 35%Ca(OH)_2_	0.02% of the 5 nm AgNPs colloidal suspension (Nano Composix, Inc., Suite K, San Diego, CA, USA) was stirred gently and sonicated with 35% Ca(OH)2 paste in a proportion of 1:1, until no lumps observed.
10 nm mixture	10 nm AgNPs (0.02%) + 35%Ca(OH)_2_	0.02% of the 10 nm AgNPs colloidal suspension (Nano Composix, Inc., Suite K, San Diego, CA, USA) was stirred gently and sonicated with 35% Ca(OH)2 paste in a proportion of 1:1, until no lumps observed.
Ca(OH)_2_ alone	35% Ca(OH)_2_ paste	Ca(OH)_2_ paste was prepared by levigating 35 gm of pure Ca(OH)_2_ powder (Somatco, Riyadh, Saudi Arabia) with 65 gm of dense water-soluble viscous vehicle of (propylene glycol: glycerin, 1:1 proportion), until a paste-like consistency was achieved. The formula permits the slow/extended ions release.
TAP	1 mg/mL TAP (metronidazole, ciprofloxacin, and minocycline)	TAP was prepared by dissolving metronidazole, ciprofloxacin, and minocycline powders (Xi’an Sgonek Biological Technology Co., Ltd., Shaanxi, China) in distilled water at a ratio of 1:1:1 (*w*/*w*/*w*) using a magnetic stirrer.

## Data Availability

The original contributions presented in this study are included in the article. Further inquiries can be directed to the corresponding author.
